# Relationship between COVID-19 vaccine hesitancy and willingness to pay for the booster dose of COVID-19 vaccine of oncology patients in Taizhou, China

**DOI:** 10.1080/07853890.2023.2165705

**Published:** 2023-02-25

**Authors:** Jia-Xiang An, Xiao-Qing Lin, Bo-Jian Xie, Tao-Hsin Tung, Jian-Sheng Zhu

**Affiliations:** a Department of Surgical Oncology, Taizhou Hospital of Zhejiang Province Affiliated to Wenzhou Medical University, Linhai, Zhejiang, China; b Department of Infectious Diseases, Taizhou Hospital of Zhejiang Province Affiliated to Wenzhou Medical University, Linhai, Zhejiang, China; c Evidence-based Medicine Center, Taizhou Hospital of Zhejiang Province Affiliated to Wenzhou Medical University, Linhai, Zhejiang, China

**Keywords:** COVID-19, willingness to pay, hesitancy, booster dose vaccine, patients with cancer

## Abstract

**Objective:**

This population-based study aimed to determine the hesitancy and willingness to pay (WTP) for the booster dose of a coronavirus disease (COVID-19) vaccine among patients with cancer in Taizhou, China.

**Patients and methods:**

A self-administered online questionnaire was administered to patients with cancer in Taizhou, China. The chi-square test, binary logistic regression model were used to evaluate the WTP for the booster dose of a COVID-19 vaccine. The minimum sample size was 218, determined by G*Power software (latest ver. 3.1.9.7). A total of 354 patients received the survey, and 256 (72.3%) patients responded.

**Results:**

Overall, 69.9% (179/256) of respondents were willing to pay for the booster dose, and 78.8% (141/179) of these patients were willing to pay 1–99 CNY. Furthermore, 50.4% (129/256) of respondents were hesitant to receive a COVID-19 vaccine. Being unhesitant was significantly associated with WTP for the booster dose (aOR: 3.040; 95% CI: 1.669–5.540).

**Conclusion:**

Hesitant patients with cancer had a lower WTP for the booster dose against COVID-19 than non-hesitant participants. These results imply that further health education programmes are essential to decrease the hesitancy of patients with cancer and enhance booster dose vaccination rates for public health improvements.

**KEY MESSAGES**
- Our research showed that 70% of patients with cancer are willing to pay for the booster dose of the COVID-19 vaccine, and most are willing to pay less than 100 CNY, and this result reflects the economic value and affordability of the third dose of vaccination.
- COVID-19 vaccine-hesitant patients with cancer had a lower willingness to pay for a booster dose against COVID-19 than non-hesitant participants and few patients are still unwilling to pay among patients do not hesitate to receive the third dose.
- Therefore, promoting willingness to pay among oncology patients and addressing vaccine hesitancy remains key.

## 1. Introduction

The unprecedented outbreak of coronavirus disease 2019 (COVID-19) has infected more than 400 million people worldwide and has resulted in the death of more than six million people worldwide [1]. These numbers continue to increase. Given that there is no effective antiviral treatment for COVID-19, the use of vaccines is important to control the epidemic [2]. In China, the full vaccination rate exceeded 85% by the end of 2021 [3]. COVID-19 has several clinical manifestations, clinical results of individuals with multiple complications and immune impairment are worse, and mortality rates owing to COVID-19 are higher [4,5].

Cancer is the leading cause of death worldwide, with 19 million new cases and 10 million deaths each year [6]. The reported incidence of malignant tumours in Taizhou, China, from 2011 to 2021 showed an overall increasing trend, and a total of 27,828 new cases of malignant tumours were reported in 2021. Patients with cancer are at high risk for severe COVID-19, particularly those receiving active cancer treatment or having metastatic diseases [7–9]. Therefore, the European Society of Medical Oncology [10], American Society of Clinical Oncology [11], and National Comprehensive Cancer Network [12] all recommend the prioritization of patients with cancer for COVID-19 vaccination unless they have contraindications. Patients with cancer usually respond well to the initial vaccination, and one may argue that the third dose should not be prioritized for them because two-dose treatments have been observed to confer protection against serious diseases. However, the immune status of patients with cancer is dynamic, and not all patients exhibit sufficient immune response [13]. Neutralizing antibody response is increased in most patients with cancer after the third vaccine dose, including in those who did not respond or were weakened after two vaccine doses [14,15]. Therefore, given that patients with cancer are generally weak and need to adhere to a strict treatment plan, the booster dose (third dose) is necessary for such patients who were under-responsive to the first two doses so that they can achieve better protection from mild diseases.

Existing literature suggests that willingness to pay (WTP) for vaccines is a key indicator of public perception and demand [16]. Therefore, the introduction of a new vaccine may require an investigation into whether the public is willing to pay for it. WTP for vaccination depends on the vaccine type and disease severity [17,18]. Hence, recognizing this important factor has become valuable for vaccination and immunization decision making. Although the booster (the third dose) of the COVID-19 vaccine remains free in China, including for high-risk patients, there will be new vaccine updates as the virus continues to mutate, and the protection of the existing vaccine will weaken against the mutated virus. In the future, it may be necessary to continue to have regular booster doses of the COVID-19 vaccine to strengthen immunity. However, health care resources are limited, and future booster vaccinations may have to be paid for. Therefore, it is important to study the willingness of different populations to pay for booster doses. Vaccine hesitancy is defined as a ‘delay in the acceptance or refusal of vaccination despite the availability of vaccination services’. COVID-19 vaccine hesitation is a complex barrier to improving vaccination rates [19,20]. Patients with cancer usually respond well to the initial vaccination, and some patients are hesitant about whether they need a third vaccine dose [13]; this hesitancy also affects patients’ WTP.

Few studies have assessed the relationship between the hesitation to receive the third COVID-19 vaccine dose and the WTP for the vaccine in patients with cancer. Thus, this study aimed to assess whether patients with cancer who are hesitant to receive the COVID-19 vaccine are willing to pay for a third COVID-19 vaccine dose and determine how much the patients are willing to pay for the third dose.

## 2. Patients and methods

### 2.1. Study design and data collection

This cross-sectional, hospital-based survey was conducted at the outpatient department of a tertiary care hospital in Taizhou, China, and the data were uploaded to Wen-Juan-Xing, one of the largest online platforms used to collect survey data in China. The participants completed a self-administered questionnaire between July 7 and August 9, 2021, by scanning a quick response code on their smartphone. The study participants were patients with cancer who had not yet received the booster dose of a COVID-19 vaccine. We used the G*Power software (latest ver. 3.1.9.7; Heinrich-Heine-Universität Düsseldorf, Düsseldorf, Germany) to determine the sample size. We used two-sided testing, Odds ratio = 2, Pr(*Y* = 1| *X* = 1) = 0.4, *α* = 0.05, power = 0.9, *R*^2^ of other independent variables (X ) = 0.5 [21,22]. The minimum sample size was computed as 218. Respondents who did not answer the survey completely were excluded. The time taken to complete the questionnaire was converted logarithmically, and if it exceeded mean ± 3SD, it was considered an outlier and was also excluded from the analysis. A total of 354 oncology patients were invited to participate in the questionnaire, and 256 questionnaires were included in the final analysis. The response rate was 72.3%.

The questionnaire employs data anonymization to ensure the confidentiality of enrollees. Oral rather than written inform consent was used. This study was approved by the Ethics Committee of Taizhou Hospital, Zhejiang Province, China (approval number: K20210705). All procedures were performed according to the guidelines of the ethics committee of the authors’ institution and adhered to the tenets of the Declaration of Helsinki.

### 2.2. Structured questionnaires and measurement

The questionnaire was a self-administered questionnaire created by the authors and uses aspects of questionnaires from our team’s previous studies [23,24]. The questionnaire comprised four parts. The first section contained questions regarding basic demographic information such as age, sex, residence, education level, occupation, history of allergic reaction to other vaccines, comorbidity and perceived risk of COVID-19.

The second section contained questions about the WTP for the booster dose of the COVID-19 vaccine and COVID-19 vaccine hesitancy. WTP for the booster dose of the COVID-19 vaccine for themselves was tested using two questions: The first question was ‘would you be willing to pay for the booster dose of COVID-19 vaccine?’ We assessed WTP by using a four-point Likert scale, where the level of reluctance increases as the score increases. A score of one indicated strongly willing, a score of two indicated willing, a score of three indicated unwilling, and a score of four indicated strongly unwilling. To facilitate analysis, the outcome was recoded as a binary variable so that the first two options were classified as ‘willing’, and the other options were classified as ‘unwilling’. Those who chose the options ‘strongly willing’ and ‘willing’ were asked the second question: ‘how much would you be willing to pay for the booster dose of the COVID-19 vaccine for yourself?’ The response options were CNY <100, 100–199, 200–299, 300–399, 400–499, and ≥500. The median WTP of individuals in China for COVID-19 vaccination is CNY 100 (USD 14.5) [25]. There was a small number of responses for CNY 100–199, 300–399, 400–499, and ≥500. On the basis of our previous studies [24], we categorized the price into two levels in the final result: CNY 1–99 and ≥100.

The third section was vaccine hesitancy, which was assessed using the following question: ‘do you hesitate to take the COVID-19 vaccine booster dose for yourself (whether you are vaccinated or not)?’ The four response options were very hesitant, hesitant, unhesitant, or very unhesitant. To facilitate analysis, the possibilities were categorized so that the first two options were classified as ‘hesitant’, and the other options were classified as ‘unhesitant’.

In addition, the fourth section involved questions on the participants’ understanding and perceptions of the efficacy and safety of the COVID-19 booster dose. Understanding about the COVID-19 vaccine was measured by the question ‘how much do you know about the COVID-19 vaccines?’ (five-point Likert scale: very well, well, relatively moderate, unknown, and not at all). Confidence in the safety of COVID-19 vaccines was assessed using a five-point Likert scale: very safe, safe, moderate, unsafe, or very unsafe. Confidence in the effectiveness of the vaccines was tested by a four-point Likert scale: great, relatively great, moderate, or little. In the final analysis, the first two options were recoded as applicable (high), whereas the other options were recoded as not useful (low) [26].

### 2.3. Statistical analysis

The primary outcome of the study was the relationship between COVID-19 vaccine hesitancy and WTP for the booster dose of the COVID-19 vaccine. First, quantity and frequency distributions were displayed for classified data. The potential factor COVID-19 vaccine hesitancy was then compared with the degree of influence by using chi-square tests. Moreover, only variables that were significant at the *p* < 0.05 in the univariate binary logistic regression model were included in the multivariate binary logistic regression model. A multivariate binary logistic regression model was used to correct for confounders and identify factors influencing the WTP for the booster dose of the COVID-19 vaccine. Logistic regression models were used to calculate the odds ratios (ORs) and corresponding 95% confidence intervals (CIs). All data were analysed using SPSS version 26.0 (IBM Corporation, Armonk, NY, USA). Differences were considered statistically significant at *p* < 0.05.

## 3. Results

Table 1 summarizes the characteristics of the patients with cancer who were surveyed. Overall, 256 questionnaires were analysed in this study. The mean age was 51.4 ± 12.8 years (range: 18–92 years). Most respondents were female (70.3%), did not have a history of allergic reaction to other vaccines (94.9%), did not have comorbidities (60.5%), were living in rural areas (62.5%), and had junior high school or a lower level of education (54.7%). Moreover, most patients did not have complete understanding about COVID-19 vaccination (54.3%). However, there was high confidence in the effectiveness of the COVID-19 vaccine (78.9%) and high confidence in the safety of the COVID-19 vaccine (69.9%). However, 50.4% of them were hesitant to receive the booster dose of COVID-19 vaccine.

**Table 1.** Demographic characteristics of the oncology patients (*N* = 256).

Independent variables	Categories	Total sample, *N*(%)/Mean ± SD
Total		256 (100%)
Sex	Male	76 (29.7%)
	Female	180 (70.3%)
Age		51.4 ± 12.8
History of allergic reaction to other vaccines	Yes	13 (5.1%)
	No	243 (94.9%)
Comorbidity	Yes	101 (39.5%)
	No	155 (60.5%)
Residence	Rural	160 (62.5%)
	Urban	96 (37.5%)
Education level	Junior high school and below	140 (54.7%)
	High school and above	116 (45.3%)
Occupation	Farmer	97 (37.9%)
	Others	159 (62.1%)
Risk perception of COVID-19	High	64 (25.0%)
	Low	192 (75.0%)
Understanding of COVID-19 vaccine	Very well	31 (12.1%)
	Well	86 (33.6%)
	Relatively moderate	111 (43.4%)
	Unknown	24 (9.4%)
	Not at all	4 (1.6%)
Confidence in the effectiveness of the COVID-19 vaccine	Great	95 (37.1%)
	Relatively great	84 (32.8%)
	Moderate,	74 (28.9%)
	Little	3 (1.2%)
Confidence in the safety of the COVID-19 vaccine	Very safe	60 (23.4%)
	Safe	142 (55.5%)
	Moderate	52 (20.3%)
	Unsafe	2 (0.8%)
	Very unsafe	0 (0%)
COVID-19 vaccine hesitancy	Very hesitant	15 (5.9%)
	Hesitant	114 (44.5%)
	Not hesitant	100 (39.1%)
	Very not hesitant	27 (10.5%)

**Figure 1.**
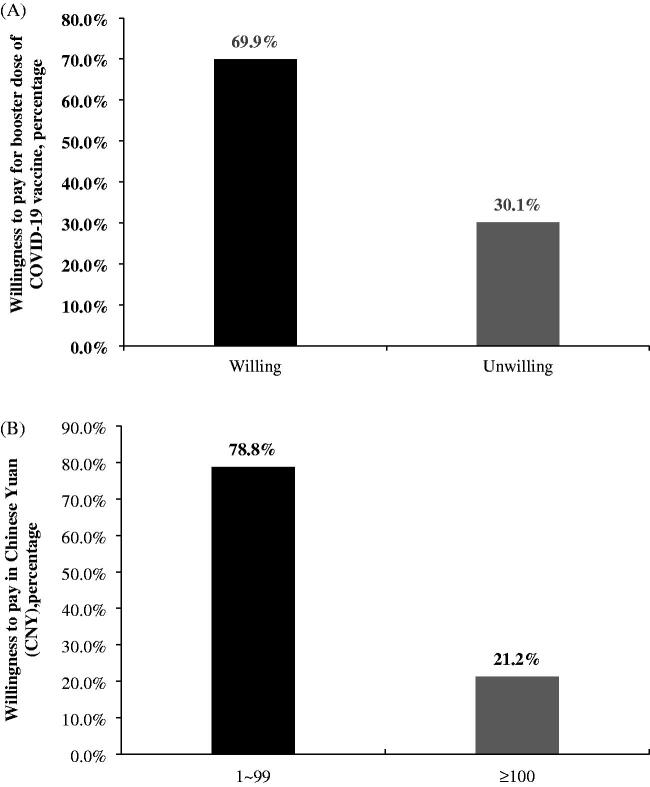
Distribution of oncology patients’ willingness to pay and the price of the COVID-19 vaccine booster. (A) Distribution of willingness to pay for the booster dose of COVID-19 vaccine (*N* = 256). (B) Distribution of prices that are acceptable to patients who are willing to pay for the COVID-19 vaccine booster (*N* = 176).

**Figure 2.**
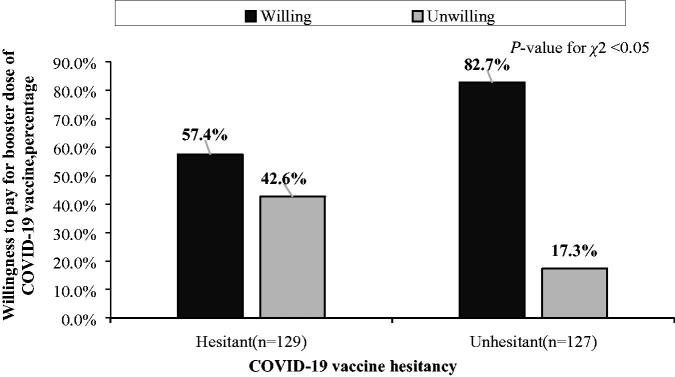
Relationship between COVID-19 vaccine hesitancy and willingness to pay for the COVID-19 vaccine booster (*N* = 256).

As shown in Figure 1, 69.9% of the respondents were willing to pay for the booster dose of the COVID-19 vaccine (A). Among those willing to pay, most of them (78.8%) were willing to pay CNY 1–99 (B). As shown in Figure 2, among oncology patients who are hesitant to receive the COVID-19 vaccine, 57.4% were willing to pay. Among oncology patients who are unhesitant to receive the COVID-19 vaccine, 17.3% were unwilling to pay. The group of unhesitant patients was significantly different from the hesitant group (*p* < 0.001).

We further calculated the degree of association between COVID-19 vaccine hesitancy and WTP for the booster dose of the COVID-19 vaccine by using a logistic regression model. As shown in Table 2, unhesitant (adjusted OR [aOR]: 3.040; 95% CI: 1.669–5.540) was significantly associated with WTP. The result suggested that those who were not hesitant about COVID-19 vaccination were 3.04 times more willing to pay for the booster dose than those who were hesitant. The adjusted *p*-value, aOR and 95% CI were adjusted for understanding of COVID-19 vaccine, confidence in the effectiveness of the COVID-19 vaccine, and confidence in the safety of the COVID-19 vaccine, which were considered significant in the univariate analyses.

**Table 2.** Logistic regression of factors associated with willingness to pay for booster dose of COVID-19 vaccine (*N* = 256).

Independent variables		Willing to pay vs. unwilling to pay			
		cOR (95%CI)	*P*_c_	aOR (95%CI)	*P*_adj_
COVID-19 vaccine hesitancy	No vs. yes	3.547 (1.992–6.317)	<0.001	3.040 (1.669–5.540)	<0.001
Understanding of COVID-19 vaccine	High vs. low	1.873 (1.078–3.253)	0.026	1.113 (0.589–2.103)	0.741
Confidence in the effectiveness of the COVID-19 vaccine	High vs. low	2.118 (1.203–3.726)	0.009	1.235 (0.610–2.498)	0.558
Confidence in the safety of the COVID-19 vaccine	High vs. low	2.749 (1.477–5.116)	0.001	1.884 (0.885–4.014)	0.101
Sex	Male vs. female	1.183 (0.653–2.141)	0.579	/	/
Age		1.014 (0.993–1.036)	0.193	/	/
History of allergic reaction to other vaccines	Yes vs. no	0.347 (0.113–1.069)	0.065	/	/
Comorbidity	Yes vs. no	0.953 (0.552–1.644)	0.863	/	/
Education level	Junior high school and below	1.171 (0.685–2.000)	0.564	/	/
	High school and above	1	1	/	/
Residence	Rural vs. urban	1.010 (0.582–1.753)	0.972	/	/
Occupation	Farmer vs. others	0.937 (0.541–1.623)	0.817	/	/
Risk perception of COVID-19	High vs. low	1.558 (0.811–2.992)	0.183	/	/

*P*_c_, cOR (95% CI): unadjusted *p*-value, crude odds ratio, and 95% confidence interval in univariate binary logistic regression model; *P*_adj_, aOR (95% CI): adjusted *p*-value, adjusted odds ratio, and 95% confidence interval in multivariate binary logistic regression model, including variables such as COVID-19 vaccine hesitancy, understanding of COVID-19 vaccine, confidence in the effectiveness of the COVID-19 vaccine, and confidence in the safety of the COVID-19 vaccine.

## 4. Discussion

Several studies have explored the attitude of patients with cancer toward COVID-19 vaccination. A cross-sectional study in France showed that 16.6% of patients with cancer were reluctant to be vaccinated at the start of the vaccination campaign [27]. In Italy, 11.2% of patients with cancer refused to be vaccinated [28]. In Poland, up to 23.46% of patients with cancer refused to be vaccinated, whereas 16.22% were undecided about whether they want to be vaccinated [29]. The vaccination willingness of patients with cancer is lower than that of the general population. A cross-sectional survey in China reported that only 8.7% of healthy adults were not willing to be vaccinated [30]. In Australia, 6% of the population refused to be vaccinated [31]. In Indonesia, 93.3% of respondents indicated that they would like to be vaccinated [32]. COVID-19 vaccine hesitation was investigated in different countries [33,34]. In the present study, 12.5% of patients with cancer refused to receive the third dose of the vaccine, whereas our previous study showed that only 8.9% of healthy respondents were unwilling to receive the third dose of the vaccine [26]. The willingness of patients with cancer to receive the third dose of vaccine is also lower than that of the general population. In a study of 2158 patients with cancer, the hesitancy rate of COVID-19 vaccination was 24.05%, and 69.09% of the patients were willing to pay for the vaccine [35]. In the current study, the hesitancy rate of patients with respect to the third dose of the vaccine was 50.4%, which was much higher than that for the first two doses. Approximately 70% of patients with cancer were willing to pay for the third dose of the vaccine, and this result is close to the WTP for the first two doses reported in the literature.

In this study, 57.4% and 42.6% of hesitant patients were willing and unwilling to pay for the third dose, respectively, and 82.7% and 17.3% of patients who were not hesitant were willing and unwilling to pay, respectively. Hesitant patients may be unwilling to pay because they are worried about the tolerance of their diseases to the vaccine. Although more patients might have the same concern, they may be more worried about the weakening of their resistance to COVID-19 owing to their condition and the increased risk of COVID-19, which explains their WTP. Although the majority of patients are not hesitant to the third dose, few patients are still unwilling to pay and are probably expecting that the third dose of the vaccine will be free, similar to the first two doses.

Current research shows that 70% of patients with cancer are willing to pay for the booster dose of the COVID-19 vaccine, and most are willing to pay less than CNY 100. This result reflects the economic value and affordability of the third dose of vaccination. To increase people’s willingness to accept and pay for the booster doses of the COVID-19 vaccine, the following interventions can be considered. First, the government can consider appropriate subsidies, especially for patients with special diseases such as cancer. Second, the cost of purchasing vaccines and making them affordable to cancer families with financial limitations can be reduced. Third, the awareness of patients with cancer about the importance of the third dose of the vaccine and their understanding of vaccination should be improved to reduce their hesitation. Cancer patients who are not hesitant about COVID-19 vaccination are 3.04 times more willing to pay for the booster dose. Vaccine hesitancy is attitudinal, and WTP is strongly correlated with payment behaviour. Some studies have shown that positive attitudes positively influence the occurrence of behaviours [36]. Therefore, promoting WTP among oncology patients and addressing vaccine hesitancy remain important. To rapidly achieve vaccine-related mass immunization, health education and publicity are still needed.

This study had some limitations. First, because this study focused only on patients in Taizhou, which is a region in China, the research results cannot be extrapolated to the whole country. Second, the study population was selected on a convenient and voluntary basis. Therefore, the results may not be representative. Third, the questionnaire did not highlight the reasons for WTP and reluctance to pay for the third dose of the COVID-19 vaccine in patients with cancer. These reasons are of great significance in guiding the formulation of targeted intervention measures and improving the practicability and comprehensiveness of the research. Fourth, we investigated the attitude of patients with cancer toward the third dose of the vaccine, but the type and number of patients with cancer involved in the study are limited. This study focuses only on subjects with commonly reported cancers, thus resulting in insufficient coverage and a relatively limited number of subjects. And we did not investigate the risk staging of patients in the questionnaire [37]. In addition, social desirability bias may occur when participants’ choices are beneficial to society. Finally, during the questionnaire survey, although the epidemic was nationwide, the COVID-19 epidemic in Taizhou was well controlled. Our estimates were taken at only one point in time and cannot be applied to the evaluation of long-term effects. The long-term trend of the WTP value of the third vaccine dose could not be determined. Further epidemiological and longitudinal investigations are essential to strengthen the findings and to better understand the willingness of patients with cancer to pay for booster doses of the COVID-19 vaccine.

## 5. Conclusion

Our results show that patients who are who are unhesitant for COVID-19 vaccine are more likely to pay for the booster dose of the COVID-19 vaccine. These findings suggest the need for further health education programs that help reduce vaccine hesitancy and increase the WTP of patients with cancer for future boosters.

## Data Availability

All data underlying the findings are within the paper. The data that support the findings of this study are available from the corresponding author, T.H.T, upon reasonable request.
